# Progression of Hepatic Adenoma to Carcinoma in the Setting of Hepatoportal Sclerosis in HIV Patient: Case Report and Review of the Literature

**DOI:** 10.1155/2016/1732069

**Published:** 2016-10-12

**Authors:** M. I. Montenovo, F. G. Jalikis, M. Yeh, J. D. Reyes

**Affiliations:** ^1^Division of Transplantation, Department of Surgery, University of Washington, Seattle, WA 98195, USA; ^2^Department of Anatomic Pathology, University of Washington, Seattle, WA 98195, USA

## Abstract

We report a case of hepatic adenoma progression to carcinoma in the setting of hepatoportal sclerosis in an HIV+ patient and provide a review of the scarce literature regarding hepatoportal sclerosis in HIV patients. We describe the clinical presentation, diagnostic workup, and management. This is the first case report in the literature of progression of hepatic adenoma to carcinoma in hepatoportal sclerosis in an HIV patient. This case also highlights the broad differential diagnosis that should always be included in the study of any liver disease in this patient population, including the performance of invasive and aggressive tests to arrive at the final diagnosis.

## 1. Introduction

Hepatocellular adenoma (HA) is a rare benign liver neoplasm that occurs most frequently in young women on oral contraceptives. It also can occur in men taking anabolic steroids or may be associated with underlying metabolic diseases, including type 1 glycogen storage disease and iron overload related to *β*-thalassemia or hemochromatosis [1]. The risks of HA include hemorrhage and malignant transformation, this last complication occurring most commonly in men.

Hepatoportal sclerosis (HPS) is one of the causes of noncirrhotic portal hypertension [2]. The hallmark histological features are seen predominantly in the portal tracts and include varying degrees of fibrosis and sclerosis of portal vein branches. Marked dilation of sinusoids may also be present [3–5]. Very rarely, patients develop hepatic synthetic compromise. None of these patients have any underlying etiology that would explain the development of this abnormality, except for case reports of HIV-infected individuals in whom histological features of HPS have been associated with prior exposure to didanosine [6–8].

Herein, we describe an HIV patient with HPS and two HAs that progressed to carcinoma and we review the current literature.

## 2. Case Report

**Figure 1:**
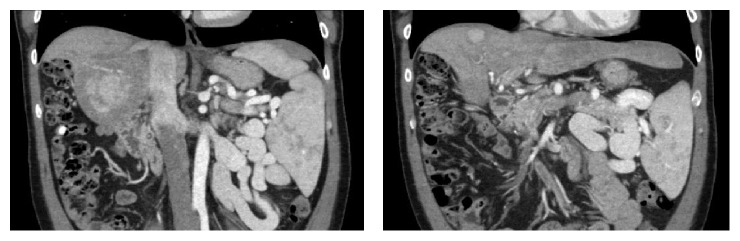
Noncirrhotic liver parenchyma but with significant portal hypertension including splenomegaly and varices.

**Figure 2:**
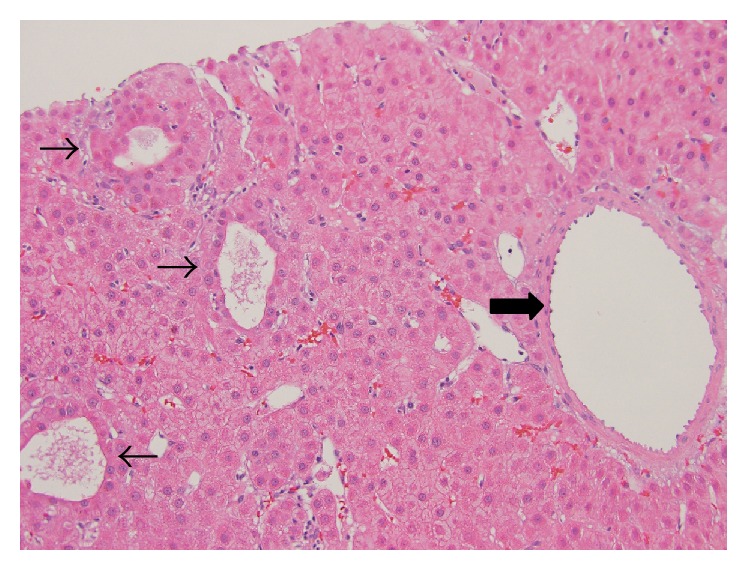
Hepatic adenoma. Well-differentiated neoplasm composed of normal appearing hepatocytes arranged in sheets and thin cords with patchy pseudoacinar growth pattern (thin arrows), scattered inflammatory foci, and bands of fibrosis with unpaired arteries (thick arrow) (H&E, 200x).

**Figure 3:**
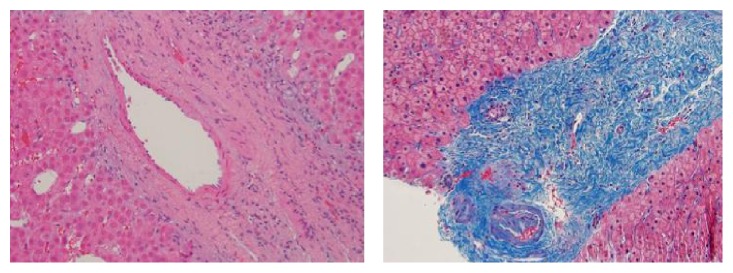
Dense portal fibrosis with loss of portal veins branches (H&E and trichrome stain, 100x).

A 48-year-old man with a 15-year history of well-controlled HIV infection presented with mild elevation of liver enzymes on regular check-up. Hepatitis panel was negative; *α*-feto protein was <5 ng/mL. Abdominal ultrasound revealed two lesions in the liver and splenomegaly. Triple-phase CT scan (Figure 1) demonstrated a noncirrhotic liver parenchyma but with evidence of significant portal hypertension including splenomegaly and varices. In addition, two liver lesions 6 cm × 5.9 cm in segment VI and 2.1 cm × 1 cm in segment VIII were identified. Both lesions had arterial enhancement, with no wash-out on venous or delayed phase, but with a pseudocapsule, most likely compatible with atypical adenoma. A targeted biopsy demonstrated a well-differentiated hepatocellular neoplasm with patchy pseudoacinar growth pattern, scattered inflammatory foci, and bands of fibrosis with occasional unpaired arteries. No reticulin framework loss was identified arguing against hepatocellular carcinoma. Immunohistochemistry for CD34 highlighted focal sinusoidal endothelialization. Immunohistochemistry demonstrated diffuse glutamine synthetase and CRP staining and patchy SAA staining which favored the diagnosis of hepatocellular adenoma, inflammatory subtype (Figure 2). Given that rare cells also showed nuclear beta-catenin staining, a beta-catenin mutation could not be entirely excluded. The nonneoplastic parenchyma demonstrated thick perivenular and portal fibrosis and lack of portal veins in focal portal tracts suggestive of HPS (Figure 3). Hepatic venous pressure gradient was 2 mmHg. The patient was on highly active retroviral therapy (HAART) and had a history of being on didanosine for 6 years.

Two years later, the patient was self-referred to our Transplant Center. MELD score was 6. Since the patient did not qualify for HCC exception points (there is no UNOS policy addressing adenomas), a right hepatectomy was offered. Liver volumes done before the surgical intervention calculated a future liver remnant of 42%. An uncomplicated open right hepatectomy was performed without any requirement of blood products. Intraoperative, significant varices and splenomegaly were identified. The postoperative course was uneventful and the patient was discharged home 4 days later. Pathology report showed two well-differentiated HCCs, margin-free, without vascular invasion. On immunohistochemistry, both tumors were positive for HepPar1 and Arginase 1 expression. Interestingly, the nonneoplastic liver parenchyma showed a normal liver parenchyma without any significant fibrosis on the trichrome stain. He has been followed up for 18 months and remains tumor-free.

## 3. Discussion

HPS is one of the several entities that are known to cause portal hypertension in the absence of cirrhosis. To date, its etiology is unknown [2]. There are increasing reports in the literature of HPS in HIV patients [6–8]. In 2010, the FDA issued an advisory regarding the potential causation of this hepatotoxicity as being due to didanosine (http://www.fda.gov/Safety/MedWatch/SafetyInformation/SafetyAlertsforHumanMedicalProducts/ucm199343.htm). This decision was based on 42 reports of noncirrhotic portal hypertension occurring in patients using didanosine. The duration of the therapy ranged from months to years. Schiano et al. [6, 7] reported the first series of HIV patients having noncirrhotic portal hypertension due to HPS. HPS may be due to an aberrant intrahepatic microvasculature as other antimetabolite medications can do, such as azathioprine resulting in hepatic venoocclusive disease. The hallmark hepatic features are found in the portal tracts, which show varying degrees of fibrosis and sclerosis of portal vein branches; marked dilation of sinusoids may also be present. In the first paper by Schiano et al. [7], the drug-induced injury resolved but the degree of HPS worsened despite the interruption of didanosine. Of note, in our case, changes of HPS were not found on the explant. We speculate that the changes of HPS might have been patchy in nature and therefore subject to sampling error. On the other hand, HPS might have completely resolved after discontinuation of the drug. The most common clinical findings are those related to portal hypertension; the hepatic synthetic function is almost always preserved. HPS might be difficult to diagnose on liver biopsy and should be considered in the differential diagnosis of patients with portal hypertension in the absence of cirrhosis. It is still unknown whether HPS is caused by didanosine or it is the HIV itself that plays the major role in the pathophysiology. Relief of the portal hypertension with surgically or radiologically placed shunts is the therapy of choice.

Hepatic adenoma is an uncommon, benign neoplasm of the liver, traditionally seen in young women of childbearing age who have a long history of estrogen-based oral contraceptive steroid use. Rarely has this been reported in men [1].

Incidental detection is due to increased use of imaging modalities for nonhepatic indications, which also contributed to the increased incidence. Diagnosis is usually made on needle core biopsies. Advances in the understanding of the molecular-genetic pathways of oncogenesis have shown that HAs are monoclonal neoplasms with unique molecular signatures which are distinct from HCCs. In 2006, a French collaborative group proposed a molecular-pathologic classification for HA, which divided these tumors into four subtypes: (1) HA with inactivating mutations of hepatocyte nuclear factor 1*α*; (2) HA with activating mutations of *β*-catenin gene; (3) inflammatory HA; (4) unclassified HA. This classification is clinically relevant because it identifies a subset of HAs with increased potential for malignant transformation (HA *β*-catenin +) [9]. Management of HAs is difficult. In 2009, Dokmak et al. reviewed their experience with resection of HAs and they concluded that only patients with HAs greater than 5 cm, inflammatory or unclassified subtype, and men have an increased risk of complications and therefore surgical resection is indicated [10].

The uniqueness of our case is twofold: (i) having the combination of HPS with two HAs on the setting of HIV and (ii) demonstrating complete remission of HPS after cessation of didanosine but without resolution of portal hypertension. Because of the inability to obtain exception points, our patient would unlikely receive a liver transplant, except from a living donor. A very extensive workup was initiated to identify the cause of portal hypertension, including a biopsy of the liver parenchyma which was diagnostic of HPS. Furthermore, we measured the hepatic vein wedge pressure which was within normal limits reassuring our presumptive diagnosis of HPS. Based on the current literature, we proceeded with resection of these lesions based on several risk factors for complications, including gender, size (>5 cm), and inflammatory subtype of adenoma. On the final pathology report, well-differentiated HCC was diagnosed along with resolution of the HPS. Even though we understand that tumor biology dictates final outcomes, without timely intervention and appropriate follow-up, there was a high chance that these tumors would have progressed to a point of being nonresectable. This case highlights the broad differential diagnosis that should always be included in the study of any liver disease in this patient population, including the performance of invasive and aggressive tests to arrive at the final diagnosis.
